# Q&A: Targeting metabolism to diagnose and treat cancer

**DOI:** 10.1186/2049-3002-2-5

**Published:** 2014-02-28

**Authors:** Ralph J DeBerardinis

**Affiliations:** 1 Children’s Medical Center Research Institute, University of Texas Southwestern Medical Center, Dallas, TX 75390, USA

**Keywords:** Cancer, Metabolism, Glutamine, Imaging, Hyperpolarisation, Isocitrate dehydrogenase, 2-hydroxyglutarate

## Interview

Dr Ralph J DeBerardinis is a paediatrician, medical geneticist and cancer researcher. He earned MD and PhD degrees from the University of Pennsylvania before a residency at the Children’s Hospital of Philadelphia. He then completed postdoctoral training with Craig B Thompson at the Penn Cancer Center where he made novel observations connecting growth factor signaling to metabolic pathways. These early studies suggested that alterations in metabolism may be a requirement for tumor growth.

He now diagnoses and treats children with inborn errors of metabolism at the University of Texas Southwestern Medical Center, where he is Director of their metabolic disease program, as well as continuing his research into cancer metabolism in the Children’s Medical Center Research Institute.

### Altered energy metabolism is now known to be a widespread feature of cancer cells. Why does a developing tumour needs to alter its metabolism?

I think it makes most sense to think about that question in the context of the acquired biological properties that separate cancer cells from non-malignant cells. Broadly speaking, cells have to acquire a number of biological properties in order to become malignant. Those features are often called the “hallmarks of cancer”, after the landmark reviews written by Hanahan and Weinberg. Those hallmarks include things like sustaining proliferative signalling, evading growth suppression and resisting cell death, all of which conspire to lead to pathologically high levels of cell survival and growth, and ultimately tumorigenesis.

All of those biological properties have to be supported by metabolic changes in order to meet the demand of those new activities. That begins to explain why cancer cells have different metabolic activities. The simplest conceptually are the ability to evade growth suppression and to sustain proliferative signalling. Growth metabolism involves the production of energy and the ability to convert whatever nutrients the cell has available to it into precursors for macromolecules. In fact, if you look at the metabolic effects of the signal transduction pathways that are most often dysregulated in cancer – particularly the PI3-kinase signaling pathway – a major role of those pathways is to provide energy and biosynthetic precursors for the cell. That is, activation of the pathway reprograms metabolism in a way that sustains growth. These pathways are often constitutively activated in cancer, so that tumor cells run the metabolic pathways that allow them to take up nutrients and convert them to macromolecules at all times.

There are other examples as well. The metabolic activities that allow a cell to resist death in the setting of a stressful microenvironment are also very important. For example, the ability to maintain energy levels when nutrients and oxygen become limiting is crucial to the health of a cancer cell. You can think about the hypoxia-induced metabolic changes that have been so well characterised over the years; and also the ability to maintain reactive oxygen species within a range that enables growth. The reactive oxygen species field is fascinating. These molecules are transient by-products of normal metabolic processes. They promote growth if present at modest levels but can trigger cell death if they become too abundant. Being able to maintain reactive oxygen species at levels that facilitate cell growth without causing cells to tip over into death pathways may be a key aspect of tumor cell metabolism.

### So reactive oxygen species have a positive and a negative effect, depending on their abundance?

Yes, there seems to be a sweet spot. A number of labs are trying to understand how cells maintain the optimal balance, and whether upsetting the balance can be used to drive tumor cells towards death.

One of the emerging themes in cancer metabolism is the surprising integration that exists between metabolic activity and signal transduction. We used to think that metabolism was fairly passive in that it was subject to the various changes in signal transduction and gene expression that allow cells to respond to extracellular cues. But over the last few years we have achieved a much greater appreciation for the fact that metabolism can also “push back”; that is, changes in metabolism elicit their own effects on signaling and gene expression networks in very interesting ways. That has been exciting. It makes some sense if you think about the fact that the mediators of these signaling processes are largely derived from intermediary metabolism. The methylation reactions that modify histone proteins and DNA – they’re derived from amino acid metabolism. There is very good evidence now that having a primary metabolic disturbance affects other dimensions of systems biology that we thought largely existed to regulate metabolism, not the reverse.

### So when you say a “metabolic disturbance” – does this link with diet, with obesity, with diabetes? Do they feed into these pathways?

Probably so. One simple example is that the acetylation state of proteins – particularly metabolic enzymes in the mitochondria – fluctuates with the feed/fast state in the liver. That may also apply to tumors characterized by very high rates of nutrient uptake. Whether these systems become deranged in the setting of more chronic conditions like obesity and diabetes is unknown, but is obviously an important area of research because of the fact that those conditions increase the risk of certain forms of cancer.

Probably the most exciting thing that’s emerged from cancer metabolism research is the fact that there are mutations in metabolic enzymes that independently influence cancer risk. Mutations in components of the succinate dehydrogenase complex in paraganglioma and pheochromocytoma; mutations in fumarate hydratase (FH) in renal cell carcinoma; and mutations in isocitrate dehydrogenase-1 and −2 (IDH1 and IDH2) that occur in a significant fraction of gliomas, acute myeloid leukemias and other forms of cancer. All of these mutations cause major perturbations in metabolism at the cellular level. The ripple effects that emanate from those metabolic perturbations impinge on signal transduction and gene expression programs in ways that appear to promote malignancy.

### The altered metabolism of cancer is to allow the proliferation of cells by generating more energy, and extra building blocks. The research that you and others have done has shown a high requirement for the amino acid glutamine in cancer. Is this a particularly important nutrient for cancer?

Yes, it seems to be, at least in some forms of cancer. The way I think about it is to ask: which features would make a nutrient most useful to a tumour cell? It should be abundant and it should be versatile. It should be able to supply a number of metabolic needs for the cell. Glutamine certainly fulfils those two criteria; it’s the most abundant circulating amino acid in humans – and it’s also versatile because of its position in the metabolic network. It can feed a very large number of pathways that promote cell survival and growth. Glutamine is a major shuttle that traffics both carbon and nitrogen between organs, meaning that it can provide precursors for basically all of the major macromolecular classes. When cells divide, they need to make proteins, nucleic acids and lipids. Glutamine feeds all of those pathways in various interesting ways. It’s also a precursor to synthesize the antioxidant glutathione and to produce NADPH, which keeps glutathione in a reduced state. Both of these are required to maintain productive reactive oxygen species levels.

Glutamine also feeds a number of other pathways that we don’t conventionally consider to be metabolic. In many cells, glutamine is the major precursor of alpha-ketoglutarate. In addition to being an intermediate in the Krebs cycle, alpha-ketoglutarate is a substrate for a large family of dioxygenase enzymes that modify proteins and DNA. This plugs glutamine into a much broader biological network than you could predict just by studying the metabolic charts. Importantly though, there are also cancer cells and tumors that quite clearly don’t consume high amounts of glutamine. We don’t entirely understand why that’s the case, except to say that in the situations where we’ve looked, the cells have found alternatives to supply those same metabolic pathways with different nutrients, particularly glucose or other amino acids.

### So it’s the versatility of glutamine – it’s a food source that can go into these multiple pathways, that make it an ideal nutrient for cancer

Yes. If you imagine a cell that’s trying to grow as rapidly as possible, that cell needs easy access to abundant nutrients that can feed a large number of pathways simultaneously. Glutamine is great for that.

### In addition to your lab work, you also are a clinician and you diagnose and treat children with inborn errors of metabolism. I wonder if you can tell us about those diseases and what we can learn from them?

I really think we can learn a lot from these diseases. Inborn errors of metabolism are genetic diseases, usually recessively inherited. They involve severe mutations in metabolic enzymes and nutrient transporters. There are many, many of these diseases involving basically every metabolic pathway. Individually, these are rare diseases, but altogether there are hundreds of them. If you add them together, they become one of the largest categories of human genetic diseases and account for a substantial fraction of inpatient hospitalization in pediatric hospitals. They provide us with unique insights into the importance of specific metabolic activities, because individuals with these diseases are analogous to knockout models for so many different components of the metabolic network.

So what we can learn from them about cancer? First, they provide an opportunity to understand the overlap between cancer and metabolic dysfunction. This is particularly true of the relatively small number of inborn errors of metabolism that are associated with an increased risk of cancer. One obvious example is a disease called (L)-2-hydroxyglutaric aciduria. This is a very rare condition where patients lack the enzyme that converts one of the enantiomers of a metabolite called 2-hydroxyglutaric acid into the Krebs cycle intermediate alpha-ketoglutarate. This metabolite, 2-hydroxyglutaric acid, or 2-HG, has generated a tremendous amount of interest recently because it is also produced in large quantities by the mutant forms of IDH1 and IDH2 that occur in gliomas, acute myeloid leukaemias and other cancers. But there are these very rare patients that have accumulations of 2-HG for a completely different reason – they lack the downstream enzyme that allows 2-HG to be converted into alpha-ketoglutarate. Amazingly enough, those patients develop brain tumors. In fact that condition is essentially the only inborn error of metabolism that’s been associated with central nervous system malignancies.

### What’s the rate of brain cancer in these patients?

It’s hard to say because the disease is so rare, but it seems to be higher than 10%.

It’s very interesting that the form of 2-HG that accumulates in the children with brain malignancies because of this metabolic disease is the (L)-enantiomer. But the opposite enantiomer – the (R)-enantiomer, accumulates in adult cancer patients with somatically acquired mutations in IDH1 and IDH2. Given the right context, it seems those two structurally related but different metabolites converge on a phenotype that gives an increased risk of malignancy, both in a child with an inborn error of metabolism and an adult with IDH1 or IDH2 mutations confined to the tumor. That will likely turn out to be an important clue about the pathogenesis of both diseases.

Conversely, we’ve learned so much about metabolic regulation now by studying cancer that it would be useful to try to use that information to better understand inborn errors. It’s quite possible that some of the agents that have been developed to try to manipulate metabolism in cancer will find a clinical home in patients with inborn errors. Certainly, the technologies that we’ve developed to monitor metabolic activity in tumors should also be useful in pediatric metabolic diseases. I’m excited about that.

### Do we see the same mutations in children with inborn errors of metabolism as we see drive altered metabolism in a developing tumor in a cancer patient?

In some cases, yes. In other cases, the metabolic disturbances are similar even if the mutations causing them are different. For example, there’s an inborn error of metabolism called glycogen storage disease type 1, which causes excessive accumulation of glycogen in the liver and kidney. The plasma of these patients contains high levels of lactate, lipids, and nucleotide metabolites, all of which you also see in cancer cells that exhibit the Warburg effect. Those patients have a long term risk of developing cancer in the liver, so it would be very interesting to understand what promotes malignancy in the setting of this metabolic disturbance.

I think the last thing to mention about patients with inborn errors in metabolism is that, as I said, these patients show us the consequences of severe loss of function of specific metabolic activities. Many of the enzymes that have been considered as targets for metabolic therapy in cancer – for example lactate dehydrogenase (LDH)-A – are mutated in inborn errors of metabolism. There are individuals with severe, whole-body LDH-A deficiency. So the question inevitably comes up, as drug companies establish programs in cancer metabolism: what toxicities do we need to worry about if we try to block LDH-A? One can study patients with the relevant disease and at least get some idea of which organ systems might be affected by the therapy.

### You’ve mentioned some potential therapeutics and I wonder whether these recent discoveries on the basic biology of cancer metabolism – of which there are now many –– have they yet changed the way we diagnose and treat patients with cancer?

Yes, they have. There’s actually a long track record of success for metabolic therapy in cancer. One of the best examples is L-Asparaginase. L-Asparaginase is an enzyme that degrades the amino acid asparagine. It’s used to treat leukemias, particularly acute lymphoblastic leukemia in children. It’s been around for decades and it is effective because very rapidly growing cells require more asparagine to support protein synthesis than they can synthesize themselves.

L-Asparaginase depletes asparagine in the plasma, starving the cancer cells. So this is really a form of metabolic therapy, and it is a big part of the reason why the cure rate in pediatric leukemias is now so high.

But more recently, I think the best example is what’s happened with the discovery of IDH1 and IDH2 mutations. These mutations were identified just about six years ago in glioma. In 2009, it was demonstrated by the team at Agios that mutations in IDH1 are associated with high levels of 2-HG in the tumor. Following this discovery, it’s become possible at several hospitals to non-invasively monitor the levels of 2-HG in brain tumors using MRI. This means that no biopsy is needed to detect high levels of the metabolite. The 2-HG levels, measured using MRI, correlate 100% with whether the tumor has a mutation in IDH1 or IDH2, again without the need to get any tissue.

### So the altered metabolism in the cancer allows you to see it using MRI?

That’s right. 2-HG is vanishingly scarce in most tissues. In tumours that have these mutations in IDH1 or IDH2, it accumulates to millimolar levels, high enough to detect by a technique called MR spectroscopy, similar to MRI. So it becomes possible to tell the patient on the day of the MRI scan whether or not there is an IDH1 or IDH2 mutation in the tumour.

That’s important for two reasons: first – because the tumor is more likely to be lower grade, that is, less aggressive, if there is a mutation in IDH1 or IDH2; and second – drugs have now been developed specifically to inhibit the mutant isoforms of IDH1 and IDH2 that occur in these tumours. So you can imagine a situation where the patient has the imaging test to detect 2-HG, then information from the scan is used to determine whether or not the patient should receive the drug. So in just six years of research, we went from identifying mutations in IDH1 and IDH2, to a sophisticated understanding of the biological consequences of those mutations, and now we have new clinical imaging strategies and new therapeutic strategies – all built around the altered metabolism. It’s very exciting, both from the perspective of new biology and because of the possibility of a new therapy for a terrible disease.

### Are there other new diagnostics and new treatments that we’ll see over the next few years based on discoveries in cancer metabolism happening today?

I think the pace is accelerating. In the imaging field, another exciting advance is a technique called carbon-13 hyperpolarisation. This is a way to image not metabolites per se but actual metabolic activity, in real time, using another MRI-related approach. This technique massively increases the visibility of labelled carbons on metabolite tracers. For example, a hyperpolarised form of pyruvate can be injected and imaged as it is converted into lactate by the tumor, giving a real-time view of the tail end of the glycolytic pathway. This approach has been used for several years in mouse models of cancer and just last year was used successfully in humans for the first time by a group at UCSF studying prostate cancer.

### Can you explain the importance of being able to measure and visualise the metabolism in the tumour?

Well, using the example of prostate cancer: these tumors are very difficult to image using other techniques. Conventional MRI is usually not sufficient and FDG PET, which is the most widely used form of metabolic imaging in cancer, is also inadequate in prostate tumors. So the hope is that hyperpolarised MRI techniques will allow oncologists and radiologists to better differentiate malignant prostate tissue from benign tissue. But there may be many other applications as well. There is a great deal of evidence that oncogenes regulate the rate of metabolic pathways, not just the abundance of metabolites. So the rate of the pathway serves as a biomarker for the effect of the oncogene, or conversely a suppressed rate may indicate that the tumor is being treated successfully. The hope is that hyperpolarisation and other new metabolic imaging techniques will substantially broaden the view of metabolism that can be obtained from live tumors. That would have great impact both on the types of research questions that can be asked in human subjects, and in the way we diagnose and monitor cancer in our patients.

## Competing interests

RD serves on the scientific advisory boards of Agios Pharmaceuticals and Peloton Therapeutics.

